# Acute Drug-Induced Hepatitis Associated With Vortioxetine: A Case Report

**DOI:** 10.7759/cureus.98067

**Published:** 2025-11-29

**Authors:** Carolina Guimarães, Rui Ribeiro, Helena Hipólito Reis, Ana Ribeiro, Jorge S Almeida

**Affiliations:** 1 Internal Medicine, Unidade Local de Saúde São João, Porto, PRT; 2 Internal Medicine, Centro Hospitalar Universitário de São João – EPE, Porto, PRT; 3 Medicine, Faculdade de Medicina da Universidade do Porto, Porto, PRT

**Keywords:** antidepressive agents, drug-induced liver injury, hepatitis, selective serotonin reuptake inhibitors, vortioxetine

## Abstract

Drug-induced liver injury (DILI) is an uncommon but potentially serious adverse reaction that can arise from the use of various antidepressants. Vortioxetine, widely used for the treatment of major depressive disorder due to its favorable efficacy and tolerability profile, has been infrequently associated with hepatic toxicity. We describe the case of a 59-year-old female who developed acute hepatitis with cholestatic features after long-term vortioxetine therapy. Extensive evaluation ruled out viral, autoimmune, and metabolic causes. Liver function improved after the discontinuation of the drug but worsened upon rechallenge, necessitating permanent withdrawal of the drug. A subsequent episode of hepatic failure after exposure to venlafaxine suggested a class-related susceptibility to selective serotonin reuptake inhibitor (SSRI)-induced hepatotoxicity. The patient recovered following cessation of antidepressants and treatment with N-acetylcysteine. This report underscores the importance of considering DILI in patients treated with vortioxetine or other serotonergic antidepressants.

## Introduction

Drug-induced acute hepatitis is an uncommon but clinically significant condition, representing a potentially reversible cause of acute liver injury that often mimics viral or autoimmune hepatitis. Prompt recognition is crucial, as delayed diagnosis may result in severe complications, including acute liver failure and the need for liver transplantation. The clinical presentation is highly variable, ranging from asymptomatic elevations in liver enzymes to jaundice, coagulopathy, and encephalopathy. Diagnosis, therefore, relies on a high degree of clinical suspicion and careful exclusion of other potential causes. In some individuals, re-exposure to agents within the same pharmacological class can trigger rapid recurrence with fulminant progression [1,2].

Vortioxetine, an antidepressant approved for major depressive disorder, has demonstrated efficacy in improving depressive symptoms, cognitive function, and overall quality of life, generally with a favorable safety profile [3,4]. Nevertheless, real-world evidence regarding its potential for hepatotoxicity is limited, as most data come from pharmacovigilance reports rather than controlled clinical trials. According to analyses of the FDA Adverse Event Reporting System, hepatobiliary adverse reactions represent only 0.33% of vortioxetine-related reports and lack detailed characterization, with no published cases of drug-induced acute hepatitis attributed to the drug [5].

In this context, we present a case of acute drug-induced hepatitis occurring shortly after exposure to two antidepressants of related pharmacological classes. This report illustrates the diagnostic challenges, underscores the importance of careful medication review, and emphasizes the need to recognize rare but potentially severe adverse reactions.

## Case presentation

A 59-year-old female patient with a history of depression and peripheral venous insufficiency was admitted after routine blood tests revealed acute hepatitis. She reported asthenia, postprandial nausea, and a 3-kg weight loss over three weeks. Laboratory investigations (Table 1) demonstrated marked cytolysis, hyperbilirubinemia, and coagulopathy. The patient also developed hepatic encephalopathy, consistent with acute liver failure. She had been taking vortioxetine for 10 weeks and denied alcohol use or exposure to other known hepatotoxic agents.

**Table 1:** Summary of laboratory findings during the first admission AST: aspartate aminotransferase; ALT: alanine aminotransferase; INR: international normalized ratio; PT: prothrombin time; ACE: angiotensin-converting enzyme

Parameter	Result	Reference range
AST	1644 U/L	<40 U/L
ALT	1987 U/L	<41 U/L
Total bilirubin	5.25 mg/dL	<1.2 mg/dL
Direct bilirubin	2.30 mg/dL	<0.3 mg/dL
INR	1.53	0.8–1.2
PT	15.8 sec	11–14 sec
Ferritin	4436.1 ng/mL	20–250 ng/mL
Transferrin saturation	75%	20–50%
ACE	79 U/L	8–52 U/L
α1-antitrypsin	172 mg/dL	100–200 mg/dL

Further workup revealed elevated iron indices with markedly increased ferritin (Table 1) but no HFE gene mutations (homozygous wild-type). Autoimmune, viral, and metabolic etiologies were excluded (Tables 2-4): α1-antitrypsin was normal, viral serologies were negative (except for prior hepatitis B contact with undetectable DNA), autoantibody screening was negative, and there was no complement consumption. Mild elevation of angiotensin-converting enzyme (ACE) was also noted. Infectious agents with hepatic tropism were excluded (Table 5).

**Table 2:** Immunological study performed during hospitalization

Auto-antibody	Result	Reference range
ANA (anti-nuclear antibodies)	Negative	<1/160
ASMA (smooth muscle antibody)	Negative	<1/20
Anti-liver antibodies (LKM-1, LC-1, SLA/LP)	Negative	-
Anti-DS-DNA (double-stranded DNA)	<10 UI/mL	<100 UI/mL

**Table 3:** Metabolic study: iron and copper metabolism studies performed

Parameter	Result	Reference range
Serum iron	152 μg/dL	49-151 μg/dL
Transferrin	215 mg/dL	200-360 mg/dL
Ferritin	982.7 ng/mL	20-250 ng/mL
Transferrin saturation	50%	20%-50%
Ceruloplasmin	29.2 mg/dL	18-45 mg/dL

**Table 4:** Viral hepatitis panel

Parameter	Result	Reference range
Anti-hepatitis A antibody – IgM	0.13	0.9-1.1
Anti-hepatitis A antibody – IgG	9.36	0.9-1.1
HBs antigen	0.2	0.9-1.1
HBc antigen	5.4	0.9-1.1
Anti-HBs antibody	332.09 UI/L	<10 UI/L
HBV DNA (viral load – PCR)	Undetectable	<10 UI/mL
HCV viral load	0.1	0.9-1.1
Hepatitis E (PCR method)	Negative	<10 UI/L

**Table 5:** Infectious agents with hepatic tropism

Parameter	Result	Reference range
Anti-HIV 1+2 antibodies	Negative	-
Anti-CMV IgM	Negative	<1.0
Anti-CMV IgG	Positive - 137 AU/mL	<10 AU/mL
Anti–Rickettsia conorii antibody	Negative	<1/64
Anti–Coxiella burnetii antibody (IgG and IgM)	Negative	<1/64

Abdominopelvic CT angiography showed a homogeneous liver of normal size without splenomegaly. Liver biopsy revealed preserved architecture with moderate portal and perivascular fibrosis, periportal lymphohistiocytic inflammation with occasional eosinophils, and evidence of previous necroinflammatory injury with ceroid-laden macrophages and acidophilic bodies. Mild iron deposition and minimal steatosis were present, without cholestasis or granulomas. The biopsy findings were nonspecific and did not confirm drug-induced injury. N-acetylcysteine therapy led to progressive improvement in cytolysis and cholestasis parameters, although mild coagulopathy persisted. Upon discharge, vortioxetine was reintroduced at a lower dose; however, mild transaminase elevation necessitated definitive discontinuation, after which gradual normalization of liver enzymes occurred.

**Figure 1:**
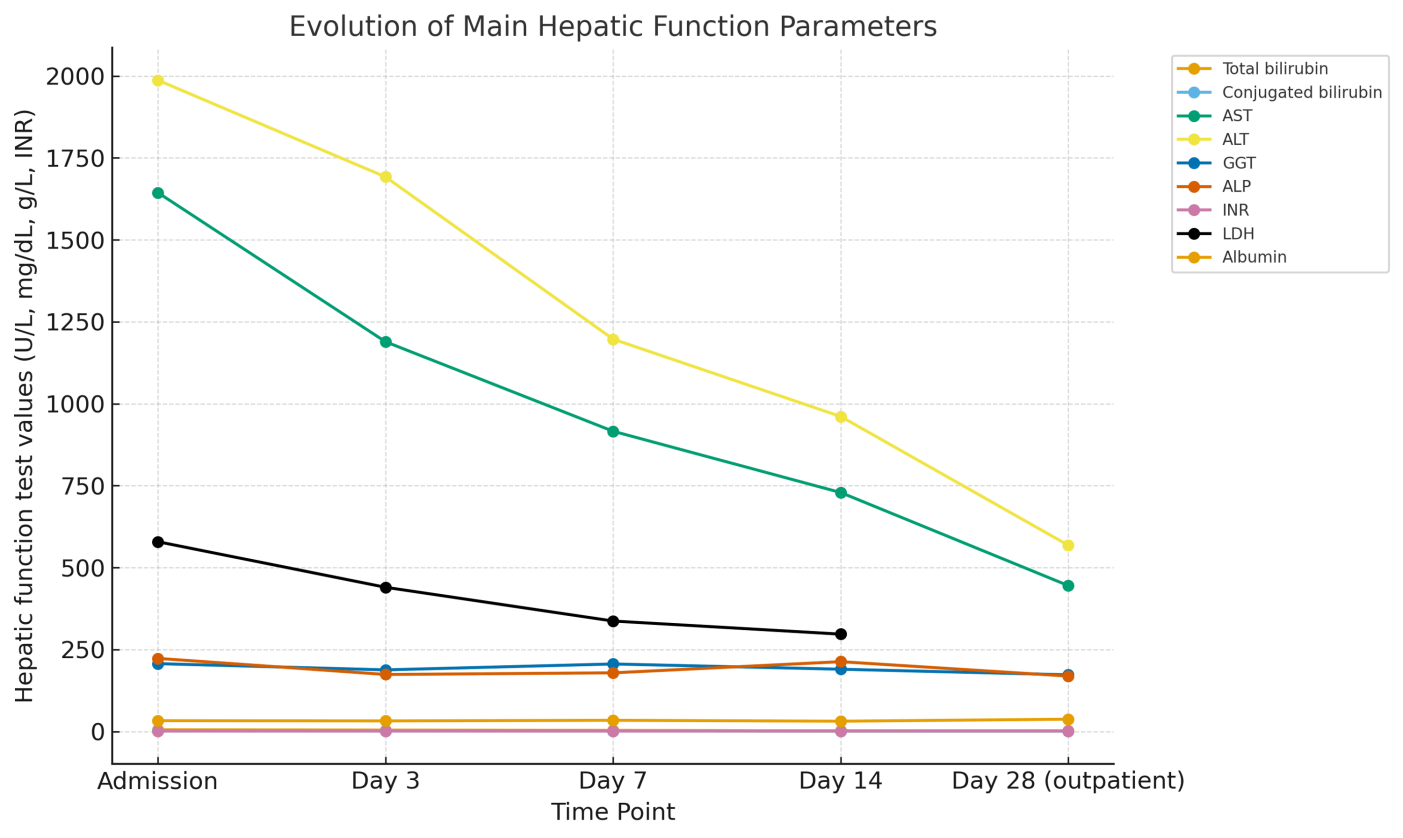
Evolution of hepatic cytolysis, cholestasis, synthesis, and excretion parameters during the second hospitalization and post-discharge follow-up AST: aspartate aminotransferase; ALT: alanine aminotransferase; GGT: gamma-glutamyl transferase; ALP: alkaline phosphatase; INR: international normalized ratio; LDH: lactate dehydrogenase

Three months later, the patient was readmitted with elevated liver enzymes and coagulopathy, but without encephalopathy, while on venlafaxine treatment. This second hospitalization reinforced the suspicion of antidepressant-related drug-induced liver injury (DILI), prompting withdrawal of the medication. N-acetylcysteine therapy was initiated, resulting in favorable clinical and biochemical evolution, with resolution of symptoms and gradual decline of hepatocellular and cholestatic markers and hyperbilirubinemia. Both the discontinuation of the antidepressant and the initiation of N-acetylcysteine occurred on day one of hospitalization, after which a progressive improvement in liver injury markers was observed, as illustrated in Figure 1.

A repeat liver biopsy, performed during the second admission, revealed a small hepatic fragment with discrete and nonspecific necroinflammatory changes and a portal tract with dense lymphocytic infiltrate, without plasma cells or polymorphonuclear cells. The limited sample precluded further characterization of the lesion, although findings were compatible with a possible exacerbation of a prior toxic or drug-induced injury.

The patient has been followed up for more than one year in the outpatient clinic, with persistently normal liver enzyme levels. The etiologic study performed during both admissions included an immunologic workup (Table 2), metabolic study (Table 3), viral hepatitis panel (Table 4), and investigation of infectious agents with hepatic tropism (Table 5).

## Discussion

The diagnosis of acute drug-induced hepatitis associated with antidepressants was established based on the temporal relationship between the described events and the initiation of two antidepressants (selective serotonin reuptake inhibitor (SSRI) and serotonin-norepinephrine reuptake inhibitors (SNRI), the histopathological findings on liver biopsy, and the absence of alternative causes identified from the clinical history, physical examination, or complementary diagnostic tests. Moreover, the initially abnormal iron studies and ACE level (Table 1) normalized upon repeat testing, and given the subsequent identification of a more plausible etiology - supported in particular by the second liver biopsy - these findings were not considered contributory to the liver injury.

DILI is mainly a diagnosis of exclusion [6,7]. Supporting this diagnosis, following antidepressant discontinuation, the patient remained asymptomatic with slow but progressive normalization of hepatic biochemical tests (Figure 1), consistent with expectations and previously reported in the literature [1,2,6]. Although a formal RUCAM (Roussel Uclaf Causality Assessment Method) score was not calculated at the time of clinical management, the patient’s course fulfilled several major RUCAM criteria - including temporal relationship, exclusion of alternative etiologies, and improvement following drug withdrawal - which supports the likelihood of antidepressant-related DILI [8].

SSRIs are widely prescribed for the treatment of depression and anxiety disorders and are generally considered safe and well-tolerated [9]. Due to the chronic nature of depression, long-term antidepressant treatment is often required. Although effective, these drugs can occasionally result in DILI [1-3,10]. It is estimated that between 0.5% and 3% of patients treated with antidepressants develop mild, asymptomatic elevations in serum aminotransferases, which are usually self-limiting [6]. Clinically apparent acute hepatitis is even more uncommon, with only isolated cases described (most notably involving sertraline) [11,12].

Establishing the diagnosis is challenging and requires careful exclusion of other potential causes, such as viral, autoimmune, and hereditary liver diseases. Another factor complicating recognition of this diagnosis is the nonspecific and heterogeneous clinical presentation, which can mimic symptoms of the underlying disease (depression) for which the medication was prescribed [7]. Some symptoms of antidepressant-induced liver injury, particularly fatigue, asthenia, anorexia, and nausea, may be misinterpreted as depressive or anxiety symptoms, potentially leading to dose escalation or even co-prescription of additional antidepressants [6].

The pathophysiology of SSRI-induced hepatotoxicity remains poorly understood [9,13]. Reported antidepressant-related hepatotoxicity cases are typically idiosyncratic - by definition not dose-dependent and without specific risk factors [14]. Nevertheless, all antidepressants have been associated with hepatotoxicity, especially in elderly or polymedicated patients [15]. Co-prescription of multiple drugs metabolized by the same cytochrome P450 (CYP450) isoenzyme pathway may increase the risk of DILI [15-17].

Comparative analyses between vortioxetine and other SSRIs in pharmacovigilance and cohort studies demonstrate a hepatotoxicity risk profile similar to that of other drugs in this class, with no significant increase in severe hepatic events [18-23]. In large European pharmacovigilance databases, the frequency of hepatic events during vortioxetine exposure was ≤1%, comparable to that observed with other SSRIs [22]. To date, no confirmed clinical cases of acute drug-induced hepatitis related to vortioxetine have been described in humans, unlike with other SSRIs [11,12]. The latency period between the initiation of this class of antidepressants and the onset of liver injury usually ranges from several days to six months [6]. Clinicopathological manifestations vary from transient enzyme elevations to fulminant hepatic failure [24-26]. The time to normalization of liver function is variable, with reports of up to six months after drug discontinuation [13,27].

Because no preventive strategies are currently available for antidepressant-induced hepatic adverse effects, early detection and prompt drug discontinuation remain essential [6,7]. In cases of suspected hepatotoxicity, the drug should be stopped while other, more common causes are excluded [6,13,16]. Although primarily established for acetaminophen toxicity, N-acetylcysteine has shown potential benefit in non-acetaminophen acute liver failure, including DILI, improving transplant-free survival in several prospective studies; this evidence supports its use in the clinical context of the present case [28]. Furthermore, patients who experience antidepressant-induced liver injury are at increased risk of recurrent hepatotoxicity if re-exposed to the same or a related antidepressant. Therefore, the offending drug should not be restarted, and other drugs with possible cross-toxicity, particularly those within the same pharmacological class, should also be avoided [29].

## Conclusions

The significance of this report stems from the fact that hepatotoxicity, and particularly acute hepatitis, are rare adverse events associated with SSRIs. Although pharmacovigilance and cohort data suggest that vortioxetine may have a hepatotoxicity profile comparable to, or possibly lower than, that of other SSRIs, such interpretations must be made cautiously, given the limitations of spontaneous reporting systems and the nature of a single case report. Notably, no prior published clinical cases of vortioxetine-associated acute drug-induced hepatitis have been reported to date. Given the widespread use of SSRIs for depression and other psychiatric disorders, continued vigilance for their rare hepatotoxic potential is essential. Early recognition and prompt discontinuation of the offending agent are key to preventing progression to severe hepatic damage and ensuring timely management.
